# Ultrasound-guided supra-inguinal fascia Iliaca compartment block for older adults admitted to the emergency department with hip fracture: a randomized controlled, double-blind clinical trial

**DOI:** 10.1186/s12877-021-02646-4

**Published:** 2021-12-01

**Authors:** Liang Chen, Yang Shen, Shuangmei Liu, Yanyan Cao, Zhe Zhu

**Affiliations:** 1 Department of Anesthesiology, Shengjing Hospital of China Medical University, Shenyang, Liaoning Province China; 2 Department of Emergency Medicine, Shengjing Hospital of China Medical University, No.36 Sanhao Street, Heping District, Shenyang, 110004 Liaoning Province China

**Keywords:** Hip fracture, Fascia iliaca compartment block, Older patients, Analgesia

## Abstract

**Background:**

Hip fracture is common in older adults, and can cause severe post-fracture pain. Fascia iliaca nerve block has consequently been used for preoperative analgesia.

**Methods:**

We performed a randomized, controlled, double-blind clinical trial and recruited older patients with hip fractures. These patients were randomized into two groups and received ultrasound-guided fascia iliaca compartment block using either the supra-inguinal approach (group F) or the classical approach (group C). Heart rate, blood pressure, and resting and exercising visual analog scales were recorded before the procedure and at 30 min, and 6, 12, and 24 h after completion of the procedure. We recorded the duration of the procedure—as well as complications such as bleeding, hypotension, and intractable vomiting; the sleep duration in a 24 h period was also documented.

**Results:**

A total of 38 patients completed the trial, and we observed no differences in the baseline characteristics or pre-procedural measurements between the two groups. Compared with the patients in group C, patients in group F exhibited significantly lower exercising VAS scores at 6 and 12 h after the procedure, faster heart rates at 6 and 24 h after the procedure, a longer procedural duration, and a longer sleep duration. There were no differences in the frequencies of complications between the two groups. The percentages of patients who took oral analgesics and the numbers of medications consumed were also not different between the two groups.

**Conclusions:**

The supra-inguinal FICB provided effective analgesia and improved exercise tolerance compared with the classical approach.

**Trial registration:**

The trial was registered at the Chinese Clinical Trial Registry (registration number: ChiCTR2100045644, registration date: 2021 April 20).

## Introduction

Hip fracture is a common fracture, accounting for more than 20% of all fractures in older patients; and with the accelerated aging of the general population, the yearly incidence of hip fractures continues to increase [1, 2]. The pain from hip fractures is intense, and in the absence of effective analgesia the pain can be exacerbated by changes in positions during physical examinations, treatments, or transportation. Severe pain may lead to an increased stress response and dramatic hemodynamic changes, which can then trigger serious cardiovascular and cerebrovascular complications such as cerebral hemorrhage and myocardial infarction. An augmented stress response also increases the risk of infection and deep-vein thrombosis. Therefore, early and aggressive applications of safe and effective analgesic treatments are essential to improving the outcomes in these patients, especially for older adults with multiple underlying diseases.

Previous studies have shown that nerve block effectively reduces the pain from hip fractures, and provides rapid-onset local analgesia that is more effective than conventional analgesia [1]. Fascia iliaca compartment block (FICB) is now being used more often for analgesia in patients with hip fractures [3, 4]. The fascia iliaca compartment is a potential cavity surrounded anteriorly by the fascia iliaca and posteriorly by the iliopsoas; and contains the femoral nerve, the obturator nerve, and the lateral femoral cutaneous nerve. The branches of the femoral nerve and the obturator nerve receive sensations from the hip joint, and the branches of the lateral femoral cutaneous nerve receive sensations from the lateral thigh. FICB can block the femoral nerve, the obturator nerve, and the lateral femoral cutaneous nerve simultaneously so as to achieve satisfactory analgesia in patients with hip fractures [5].

An ultrasound-guided supra-inguinal approach has in recent years been proposed as a novel method for performing FICB. Compared with the classical approach, the supra-inguinal approach directs the puncture needle cephalad, allowing for easier diffusion and improved analgesic effects from only a small amount of medication [6, 7]. However, only a limited number of studies exist on the analgesic effects of supra-inguinal FICB in the early stages of fracture, especially in the older patient. Therefore, we herein performed a randomized, controlled, double-blind clinical trial to evaluate the early analgesic effects of ultrasound-guided supra-inguinal FICB in older patients who present with hip fractures in the emergency department.

## Materials and methods

### Study design and participants

This study was approved by the Institutional Review Board of Shengjing Hospital of China Medical University, Shenyang, China (approval number: 2021PS511K, approval date:12/05/2021). Letters of informed consent signed by patients were obtained. This study was conducted in accordance with the principles of the Declaration of Helsinki. The trial was registered at the Chinese Clinical Trial Registry (registration number: ChiCTR2100045644, registration date: 20/04/2021).

This study was conducted at Shengjing Hospital from April to July 2021. We performed a randomized, controlled, double-blind clinical trial on patients enrolled from our emergency room. The inclusion criteria were patients 65 years of age or older with acute hip fracture. The exclusion criteria were 1) having experienced other severe trauma, 2) manifesting coagulopathy or receiving anticoagulants, 3) currently on chronic analgesics, 4) a history of allergy to local anesthetics, 5) an infection in the area where FICB was to be performed, and 6) having significant visual, hearing, or cognitive impairment.

### Study protocol

All study participants were randomly assigned to either the supra-inguinal approach group (group F) or the classical approach group (group C), and baseline characteristics were collected. After the patients arrived in the emergency room, they underwent intravenous catheterization, and a cardiac monitor was used to record blood pressure, heart rate, and oxygen saturation. FICB was attempted after preparation of the necessary resuscitative medications and equipment.

Patients in group F underwent the following steps. 1) With the patient in the supine position, the inguinal area was disinfected and a sterile ultrasound probe was placed next to the inguinal ligament with its long axis parallel to the ligament. The femoral artery and the femoral nerve were clearly identified. (2) The probe was then moved laterally to localize the sartorius muscle. The image of the sartorius muscle was placed at the center of the screen and traced cephalad until its disappearance with the movement of the probe to the hypoechoic anterior superior iliac spine. The ultrasound probe was rotated 90° to identify the anterior superior iliac spine, the iliac muscle, and the abdominal muscles on the screen. The needle was then inserted from the caudad side and directed cephalad using an in-plane technique, and the needle tip was allowed to puncture the fascia iliaca to reach the fascia iliaca compartment. (3) After confirmation of the correct needle-tip placement by injecting 5 mL of saline, we administered 30 mL of 0.2% ropivacaine. The fascia iliaca was then separated from the iliac muscle as the medication diffused. (4) Finally, the needle was removed, and the puncture point was dressed with a sterile bandage after compressing the site for 1 min to stop bleeding.

Patients in group C underwent the same steps as described in 1, 3, and 4 above. However, in step 2, after localizing the fascia lata, fascia iliaca, and iliopsoas muscle that were lateral to the femoral nerve, a disposable sterile needle was inserted laterally and pointed toward the inner side. Then, the tip of the needle punctured the fascia iliaca to reach the fascia iliaca compartment. After the procedure, patients received 50 mg of flurbiprofen injection every 12 h. Oral oxycodone and acetaminophen tablet was administered if the patient complained of severe pain.

### Outcome measurements

The procedural durations were recorded, and sensory blocks were evaluated 30 min after the completion of FICB. An effective block was defined as the loss of pinprick sensation in the innervated areas of the femoral, lateral femoral cutaneous, and obturator nerves. The areas evaluated included the anterior thigh for the femoral nerve, the lateral thigh for the lateral femoral cutaneous nerve, and the inner thigh for the obturator nerve.

We recorded pain intensities in both groups—as well blood pressure and heart rate—at the following timepoints: before the procedure (T0) and at 0.5 (T1), 6 (T2), 12 (T3), and 24 h (T4) after the procedure. Pain intensity was assessed using the visual analogue score (VAS) on a scale of 0–10, with a higher score corresponding to more severe pain. These measurements were first recorded at rest and then repeated with the affected limb externally rotated to 15°.

Complications within 24 h after the procedure—including bleeding from the puncture site, local anesthetic overdose, nausea and vomiting, and hypotension (defined as a reduction in > 20% from the baseline blood pressure)—were recorded. The amounts of oxycodone and acetaminophen tablets taken and sleep duration were also documented.

Entering the trial did not affect the patients’ operating time. For those patients who had completed adequate preoperative preparation, we supported patients in undergoing surgery as soon as possible. Because surgery would interrupt follow-up, we excluded patients who underwent surgery within 24 h after the FICB procedure.

All methods were performed in accordance with the relevant guidelines and regulations, there are no deviations from the study protocol approved by the Institutional Review Board of Shengjing Hospital of China Medical University.

### Statistical analyses

#### Sample-size analysis

We used the visual analogue score (VAS) after the intervention as the main observation index. Based on previously published research, we preset the VAS score of F group as 3, C group as 4, and the standard deviation as 1. We believed that reducing the VAS score by 1 would have clinical significance. To achieve 90% statistical power (β = 0.1) with a two-sided confidence interval of 95% (α = 0.05), we used PASS11 to calculate that 18 patients should be recruited to each group. Considering the possible loss of follow-up or withdrawal of patients, we requested the review committee to allow a 30% increase of patients. The final sample size for the planned recruitment was a total of 50 patients.

#### Randomization and blinding

Patient randomization was performed by SPSS software (IBM Corp., Armonk, NY, USA). The researchers used SPSS to generate 50 groups of random numbers, set the smaller 25 numbers as the experimental group and the larger 25 numbers as the control group, and then hid the grouping results in a series of sealed envelopes with the patient number written on the outside. The envelope was placed in the emergency treatment room. When the test patient entered the treatment room, the doctor implementing FICB opened the envelope corresponding to the patient’s number so that the patient was assigned to the corresponding group and given the corresponding FICB operation. The randomized doctors were only responsible for statistical analysis in the next trial. During the trial, only the doctor who implemented FICB knew the grouping of the patients, and they did not participate in other parts of the trial. The doctors responsible for follow-up and data collection did not know the grouping of the patients. The patients do not know what kind of FICB they received, nor did they know their grouping.

#### Statistical analysis

SPSS 24.0 software (IBM Corp., Armonk, NY, USA) was used for statistical analysis. We first tested continuous data normality, and data that followed a normal distribution are presented as means ± standard deviation (x ® ± s) and compared using multivariate ANOVA (inter-group comparisons) or repeated-measures ANOVA (intra-group comparisons). Data from a non-normal distribution are presented as medians with an interquartile range (IQR) and compared with the Mann-Whitney U test. Categorical data are presented as numbers and percentages and analyzed by Chi-squared analysis or Fisher exact-probability test. A *P* < 0.05 was considered statistically significant.

## Results

**Fig. 1:**
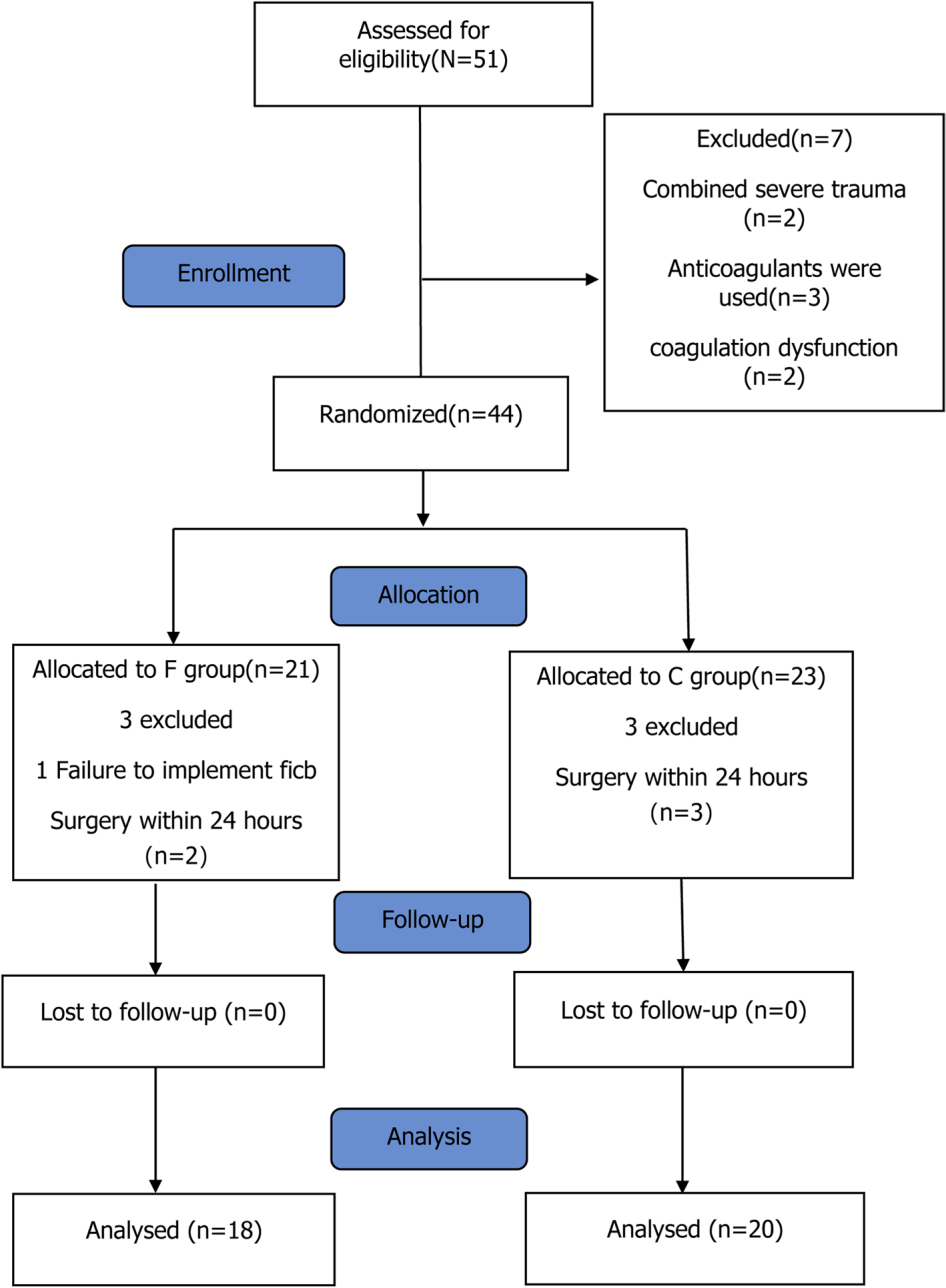
Flow diagram of the study

We interviewed 51 patients, and seven patients were excluded from the study—two with severe trauma, three on anticoagulants, and two with coagulopathy. During the clinical trial, one patient failed the FICB and five patients underwent the surgical operation within 24 h of the FICB; for a total of 38 patients who completed this clinical trial. The CONSORT flow chart is shown in Fig. 1, and baseline characteristics were comparable between the two groups (Table 1).

**Table 1:** The comparison of the patient characteristics between 2 groups

	F group(*n* = 18)	C group (*n* = 20)	test	*P* value
Gender (male / female)	6/12	6/14	X^2^ = 0.049	0.825
Age (years)	72.1 ± 6.0	73.8 ± 5.4	t = −0.939	0.354
Height (cm)	163.8 ± 4.6	164.9 ± 4.5	t = −0.755	0.455
Weight (kg)	60.3 ± 8.7	58.4 ± 7.3	t = 0.763	0.450

The duration of the procedure was significantly longer in group F relative to group C (10.5 ± 1.5 vs. 9.5 ± 1.2 min). The femoral nerves were successfully blocked in all patients of both groups. Compared with the patients in group C, more patients in group F experienced successful blockage of their lateral femoral cutaneous nerves and obturator nerves. The above results are shown in Table 2.

**Table 2:** Comparison of duration of the procedure and success ratio of nerve block between two groups

	F group (*n* = 18)	C group (*n* = 20)	test	*P* value
duration of the procedure (min)	10.5 ± 1.5	9.5 ± 1.2	t = 2.248	0.031
femoral nerve (effective/total)	18/18	20/20		
lateral femoral cutaneous nerve (effective/total)	15/18	10/20	F = 4.677	0.043
obturator nerve (effective/total)	12/18	5/20	F = 6.653	0.021

Analysis of VAS showed statistically significant lower- exercising VAS scores at T2 and T3 timepoints in group F compared with group C. Otherwise, there were no statistically significant differences in the resting VAS scores or the exercising VAS scores at other timepoints between the two groups. The above results are shown in Table 3.

**Table 3:** Comparison of resting and exercising VAS scores between the two groups at different time points

	Resting VAS	Exercising VAS
F group (*n* = 18)		
T0	5.5 ± 1.0	7.6 ± 0.9
T1	3.0 ± 0.7	3.5 ± 0.5
T2	2.7 ± 0.7	3.4 ± 0.9 a*
T3	2.9 ± 0.7	3.6 ± 0.9 b*
T4	3.8 ± 1.1	4.4 ± 0.9
C group (*n* = 20)		
T0	5.2 ± 1.1	7.5 ± 1.1
T1	3.3 ± 0.8	3.9 ± 0.8
T2	2.9 ± 0.6	4.1 ± 0.6 a*
T3	3.3 ± 0.6	4.3 ± 0.8 b*
T4	3.6 ± 0.7	4.5 ± 0.8

Notes

*VAS* visual analogue scale

^a^ANOVA, F = 6.448, *p* value = 0.016

^b^ANOVA, F = 5.794, *p* value = 0.021

^*^P < 0.05, there was significant difference between the two groups

Compared with group C, group F exhibited statistically lower heart rates at the T2 and T4 timepoints; but there were no differences in the heart rates at the T0, T1, or T3 timepoints between the two groups. There were also no differences in the mean arterial pressure at any timepoint between the two groups. The above results are shown in Table 4.

**Table 4:** Comparison of MAP and HR between the two groups at different time points

	MAP (mmHg)	HR (bpm)
F group (*n* = 18)		
T0	107.1 ± 10.3	81.6 ± 4.7
T1	106.8 ± 10.6	75.2 ± 5.6
T2	96.9 ± 8.1	70.4 ± 3.7a*
T3	101.2 ± 9.5	72.9 ± 5.7
T4	103.2 ± 10.9	73.1 ± 4.8b*
C group (*n* = 20)		
T0	108.2 ± 10.6	82.3 ± 6.3
T1	107.4 ± 8.5	76.7 ± 5.2
T2	99.4 ± 8.9	73.2 ± 3.4a*
T3	101.8 ± 8.8	73.6 ± 6.8
T4	106.5 ± 11.3	76.4 ± 5.2b*

Notes

*MAP* mean arterial pressure, *HR* heart rate

^a^ANOVA, F = 5.659, *p* value = 0.023

^b^ANOVA, F = 4.157, *p* value = 0.049

^*^P < 0.05, there was significant difference between the two groups

Within 24 h of the procedure, patients in group F (median, 7.5; IQR, 7, 8 h) showed a significantly longer sleep duration than the patients in group C (7; IQR 6, 7.5 h). There were no differences in terms of the proportions of patients who took oxycodone and acetaminophen (22.2% in group F vs. 30% in group C), or the average numbers of tablets consumed between two groups (1.5, IQR 1, 2 in group F vs. 1, IQR 1, 2 in group C). Nausea and vomiting were experienced by one patient in group F and by two patients in group C, with no statistically significant difference between the groups. There was no bleeding at the puncture site, local anesthetic overdose, or hypotension reported for either group.

## Discussion

In the present study we showed that ultrasound-guided supra-inguinal FICB provided effective analgesia in older patients with hip fractures. Compared with the classical approach, the supra-inguinal approach achieved a higher likelihood of success in blocking the lateral femoral cutaneous nerve and the obturator nerve, and therefore provided better analgesic effects during movement.

A common practice in Chinese emergency rooms that is used to control pain in older patients with hip fractures is to prescribe non-steroidal anti-inflammatory drugs (NSAIDs) or opioids such as morphine or pethidine. NSAIDs usually provide inadequate analgesia and carry the risk of peptic ulcers and renal failure. Opioids can provide better analgesia but carry complications such as nausea, vomiting, constipation, urinary retention, hypotension, and respiratory depression [8–10]. Pain, nausea, and vomiting often affect the quality of sleep, which can then cause further anxiety and decrease the quality of health care while increasing the cost of medical expenses.

FICB has been accepted for use in preoperative analgesia with respect to patients with hip fractures [11, 12]. FICB can provide safe and effective pain relief, especially for analgesia during movement, and can also reduce the requirement for opioids [13]. However, the classical approach of FICB has its inherent disadvantages. The local anesthetic may not spread well in the cephalad direction, which might result in incomplete block of the obturator nerve [12, 14, 15]; and effective block of the obturator nerve is always under investigation with regard to FICB. Although local anesthetic injection under the fascia iliaca can block the obturator nerve through diffusion, the analgesic effect might be inadequate due to the large distance between the obturator nerve and the femoral nerve [16, 17]. Zhou et al. concluded that the analgesic effect of the supra-inguinal approach for FICB on the femoral and obturator nerve blocks was better than that for the classical approach in patients with hip fractures. These authors postulated that the inferior effect with the classical FICB approach was due to incomplete block of the obturator nerve in patients [18].

A novel approach to performing supra-inguinal FICB has in recent years been introduced in the clinic, and this uses the anterior superior iliac spine as a bony landmark to identify the fascia iliaca and iliac muscle [6, 7]. In this approach, the direction of the puncture needle is oriented toward the patient’s head, which makes it easier for the analgesic to diffuse cephalad and to apply a more complete block of the obturator and lateral femoral cutaneous nerves. Investigators reported in a clinical trial that supra-inguinal FICB provided effective analgesia in patients over 65 years of age after hip-replacement surgery, which could then result in less opioid consumption than with classical FICB [19]. Another study also showed that the supra-inguinal approach provided a more adequate block of the lateral femoral cutaneous nerve compared with the classical approach [20]. In a cadaver study, Kris Vermeylen et al. reported that 40 mL of local anesthetic was required to stain the femoral, lateral femoral cutaneous, and obturator nerves in adult cadavers by supra-inguinal FICB [21]. The study by Kumiko et al. showed that the EV95 of 0.25% ropivacaine in supra-inguinal FICB for blocking all three nerves was 27.0 mL [22]. We used 30 mL of analgesic in our clinical trial and demonstrated that the supra-inguinal approach was superior to the classical approach when evaluating sensory block in the obturator and lateral femoral cutaneous nerves.

Exercising VAS in our study was lower in group F than in group C at 6 and 12 h after the block, with no differences at other timepoints. Our explanation for this was that it might take time for the analgesic to spread and take effect. By 0.5 h after the procedure, the analgesia began to display differential effects on the cutaneous branch—but not the motor branch—of the obturator nerve. And by 6–12 h after the procedure, the supra-inguinal approach exerted a more potent block on the obturator nerve, which resulted in lower exercising VAS scores.

There was no difference in the resting VAS between the two groups, and our explanations for this follow. 1) The obturator nerve plays a limited role at rest, with a study showing that the block of the obturator nerve did not relieve pain after total hip arthroplasty [23]; this suggests that the obturator nerve was less important than the femoral nerve in analgesia during hip fractures. 2) There might be errors in the assessment of the obturator nerve block, as accurate assessments have proven quite difficult due to significant variation in the cutaneous distribution of the obturator nerve. For example, it was reported that the obturator nerve provided no cutaneous innervation in 57% of the population [24]. Although an accurate evaluation of the obturator nerve requires assessment of adductor muscle strength [25], this method could not be used in our patients due to the limited movement capabilities of their lower extremity with hip fracture.

We observed no statistical difference in the number of patients who took acetaminophen or the amount of medication used between the two groups. Interestingly, the patients in group F possessed a longer sleep duration, which suggested a better overall analgesic experience of the patients in this group. However, this conclusion was not consistent with the results of comparable measurements on the VAS, mean arterial pressure, or heart rate between the two groups. Sleep is a subjective measurement and might be affected by a minimal change in pain sensation. A longer sleep duration might even indicate that the supra-inguinal approach made patients more comfortable and exerted an overall benefit in improving postoperative recovery and overall outcome.

The heart rate in group F was lower than that in group C at 6 and 24 h after the procedure. However, the difference of mean value may not have clinical significance. This difference in the heart rate could not be explained by the different analgesic effects between the two groups, as there was no difference in the resting VAS scores between the groups at these timepoints. We posited that the most likely reason for this was sleep-related, since patients were asleep by 6 h after the procedure when their initial biochemical and imaging examinations had been completed. Patients in group F might have experienced a deeper sleep, which resulted in a slower heart rate. By 24 h after the procedure, the longer sleep duration in patients in group F might have allowed them to have a slower heart rate.

In the present trial our supra-inguinal approach was of longer duration than the classical approach, although there was no significant difference. The supra-inguinal approach required more technical skill, as well as a better understanding of the local anatomic structures and spatial orientations. We realized at the final step of the procedure that color Doppler images should be applied to find the deep circumflex iliac artery, which would indicate the correct placement of the ultrasound probe. This would also avoid accidental injection of analgesic directly into the vessels.

Nerve block is presently used infrequently in the emergency room [26, 27], which is likely due to a lack of understanding of the relevant technique and skills involved, the short stay in the emergency room, and the change in the physician-in-charge.

The limitations to our study included not assessing muscle strength after the FICB, since our patients were unable to move the affected hip. We thereby only selected patients who did not undergo surgery within 24 h of the FICB procedure, and this might have caused selection bias.

## Conclusions

Ultrasound-guided supra-inguinal FICB furnished early and effective analgesia, improved exercise tolerance, and administered better sleep quality to older patients presenting with hip fractures in the emergency room.

## Data Availability

The raw data has been uploaded to the database. The public access to the database is open. DOI: 10.6084/m9.figshare.15000408 The datasets used and/or analysed during the current study are available from the corresponding author on reasonable request.

## Abbreviations

FICB: Fascia iliaca compartment block
VAS: Visual analogue score
MAP: Mean arterial pressure
HR: Heart rate
NSAIDs: Non-steroidal anti-inflammatory drugs
