# Correction to: Ultrasound-guided supra-inguinal fascia Iliaca compartment block for older adults admitted to the emergency department with hip fracture: a randomized controlled, double-blind clinical trial

**DOI:** 10.1186/s12877-021-02698-6

**Published:** 2022-01-03

**Authors:** Liang Chen, Yang Shen, Shuangmei Liu, Yanyan Cao, Zhe Zhu

**Affiliations:** 1 Department of Anesthesiology, Shengjing Hospital of China Medical University, Shenyang, Liaoning Province China; 2 Department of Emergency Medicine, Shengjing Hospital of China Medical University, No.36 Sanhao Street, Heping District, Shenyang, 110004 Liaoning Province China

**Correction to: BMC Geriatr 21, 669 (2021)**

**https://doi.org/10.1186/s12877-021-02646-4**

After publication of this article [1], the authors reported reference [24] was missing and should have been: [24] Bouaziz H, Vial F, Jochum D, Macalou D, Heck M, Meuret P, et al. An evaluation of the cutaneous distribution after obturator nerve block. Anesth Analg. 2002;94(2):445-9.

The original article [1] has been updated.

## References

[1] Chen L, Shen Y, Liu S (2021). Ultrasound-guided supra-inguinal fascia Iliaca compartment block for older adults admitted to the emergency department with hip fracture: a randomized controlled, double-blind clinical trial. BMC Geriatr.

